# Chemogenomic approach to increase accuracy of QSAR modeling of inhibition activity against five major P450 isoforms

**DOI:** 10.1186/1758-2946-5-S1-P23

**Published:** 2013-03-22

**Authors:** Sergii Novotarskyi, Iurii Sushko, Robert Koerner, Igor V Tetko

**Affiliations:** 1 eADMET GmbH., Munich, 85764, Germany; 2 Institute of Structural Biology, HelmholzZentrum Muenchen, Munich, 85764, Germany

## 

Cytochromes P450 (CYP) are a superfamily of enzymes, involved in metabolism of xenobiotic compounds. CYP are involved in metabolism of a large amount of drugs, currently present on the market. Therefore, prediction of CYP inhibition activity of small molecules poses an important task, especially in early stage drug discovery, due to high risk of drug-drug interactions. It is estimated that CYP enzymes metabolize over 75% of currently marketed drugs. Of these reactions over 90% are facilitated by CYP1A2, CYP2C9, CYP2C19, CYP2D6 and CYP3A4. This makes these enzymes particularly interesting targets for in-silico inhibition prediction. Accurate prediction of inhibition activity of small molecules against CYP enzymes is particularly important in the field of personalized medicine discovery.

High promiscuity with respect to substrates of the studied cytochromes limits the approach of traditional QSAR methods. Including structural information of the protein is crucial to obtaining predictive models. In this work the modeling is performed on a set of chemogenomic descriptors obtained from protein-ligand complexes. The quality of the descriptors is benchmarked in QSAR modeling of HTS data for human CYP450 inhibition. The calculation of descriptors involves a flexible docking of the molecule to the rigid binding cite of the cytochrome (in this study the AutoDock Vina tool was used). The obtained top-ranked conformation is then processed to obtain the descriptors.

The training sets for the benchmarked models were obtained from PubChem BioAssay database (assays AID410, AID883, AID899, AID884 and AID891 for CYP1A2, 2C9, 2C19, 3A4 and 2D6, respectively). The test sets are obtained from the AID1851 assay by excluding all molecules present in the training set.

The models presented in the study achieved 82 - 87% of correctly classified compounds on the validated training set and 65 - 75% of correctly classified instances on the test sets. The dramatic difference in model performance between the test and the validated training sets can be explained by structural dissimilarity of the sets.

The use of applicability domain approaches to select only confident predictions allowed to achieve the accuracy of 90% of correctly classified instances on the subset of 20% most confident predictions of the test set.

The datasets and the benchmarked models are available on the Online Chemical Modeling Environment (http://ochem.eu).

